# Mycosis Fungoides and Sézary Syndrome: Microenvironment and Cancer Progression

**DOI:** 10.3390/cancers15030746

**Published:** 2023-01-25

**Authors:** Gabor Dobos, Ingrid Lazaridou, Adèle de Masson

**Affiliations:** 1 Charité—Universitätsmedizin Berlin, Corporate Member of Freie Universität Berlin and Humboldt, 10117 Berlin, Germany; 2 Department of Dermatology, Universität zu Berlin, 10117 Berlin, Germany; 3 Dermatology, Brown University, Providence, RI 02912, USA; 4 Dermatology Department, Saint-Louis Hospital, AP-HP, 75010 Paris, France; 5 Department of Medicine, Université Paris Cité, 75010 Paris, France

**Keywords:** mycosis fungoides, Sézary syndrome, microenvironment, cutaneous T-cell lymphomas

## Abstract

**Simple Summary:**

The surrounding cells of the tumor tissue are called microenvironment, and they may contribute to disease progression and/or antitumor immune response. Much has been learned about the microenvironment of mycosis fungoides and Sézary syndrome over the past decades and how it is affected by treatments. This review summarizes the recent advances in the field.

**Abstract:**

Mycosis fungoides and Sézary syndrome are epidermotropic cutaneous lymphomas, and both of them are rare diseases. Mycosis fungoides is the most frequent primary cutaneous lymphoma. In about 25% of patients with mycosis fungoides, the disease may progress to higher stages. The pathogenesis and risk factors of progression in mycosis fungoides and Sézary syndrome are not yet fully understood. Previous works have investigated inter- and intrapatient tumor cell heterogeneity. Here, we overview the role of the tumor microenvironment of mycosis fungoides and Sézary syndrome by describing its key components and functions. Emphasis is put on the role of the microenvironment in promoting tumor growth or antitumor immune response, as well as possible therapeutic targets. We focus on recent advances in the field and point out treatment-related alterations of the microenvironment. Deciphering the tumor microenvironment may help to develop strategies that lead to long-term disease control and cure.

## 1. Introduction

**Figure 1:**
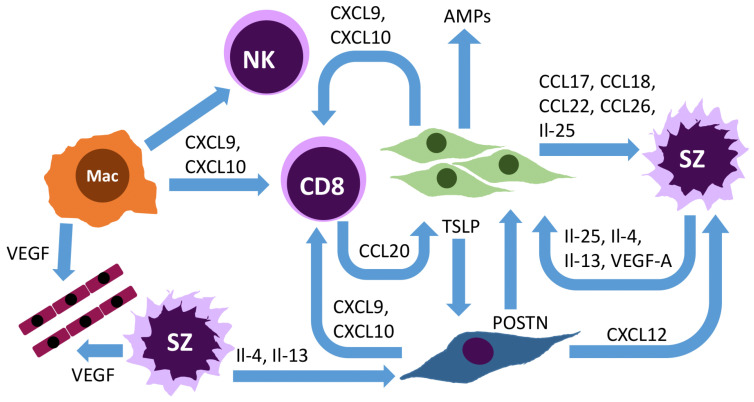
Interactions in the tumor microenvironment in mycosis fungoides and Sézary syndrome. The figure shows selected interactions in the microenvironment of mycosis fungoides. Cells in orange represent macrophages, green keratinocytes, blue fibroblasts, red endothelial cells, cells marked with SZ represent tumor cutaneous T-cell lymphoma cells (Sézary cells), CD8 cytotoxic lymphocytes, and NK natural killer cells. Abbreviations: AMP—antimicrobial peptide, CCL—chemokine ligand, CXCL-chemokine X ligand, Il—interleukin, POSTN—periostin, TSLP—thymic stromal lymphopoietin, VEGF—vascular endothelial growth factor.

**Figure 2:**
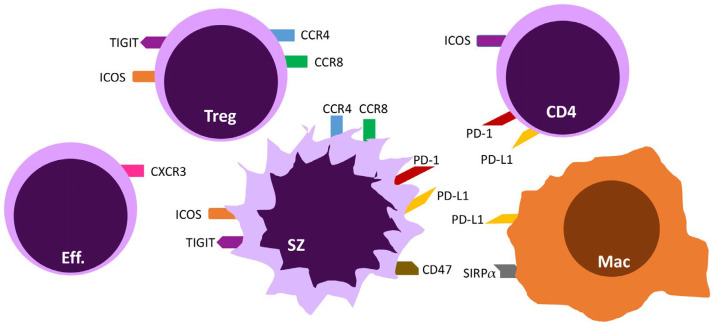
Main targetable immune checkpoints in mycosis fungoides and Sézary syndrome. The figure shows selected targetable immune checkpoints in mycosis fungoides and Sézary syndrome. Abbreviations: Eff-effector lymphocyte, Treg—regulatory T-cell, SZ—tumoral cell (Sézary cell), CD4—benign helper T-cell, Mac—macrophage, CCR—chemokine receptor, CXCR—chemokine X receptor, ICOS—inducible costimulatory receptor, PD-1—programmed death 1, PD-L1—programmed death ligand-1, SIRPα—signal regulatory protein α, TIGIT—T cell immunoreceptor with Ig and ITIM domains.

Mycosis fungoides is the most frequent subset of primary cutaneous T-cell lymphomas, and it represents approximately 60% of them. It usually presents with erythematous, scaly, or hypopigmented patches or plaques in the early stage and with tumors or erythroderma in the advanced stage. The nodal or visceral disease may occur in late-stage mycosis fungoides. Sézary syndrome is an advanced-stage cutaneous T-cell lymphoma subtype characterized by blood involvement and erythroderma (erythema over the whole-body surface area). Mycosis fungoides patches and plaques usually comprise a minority of neoplastic T cells [1], most of the infiltrate being made of reactive T cells and other cell types such as macrophages, fibroblasts, dendritic cells (DCs), mast cells, myeloid-derived suppressor cells (MDSCs), and others (Figure 1). The tumor microenvironment has emerged as a critical player in the pathogenesis of tumor progression and, in particular, in mycosis fungoides and Sézary syndrome [2,3,4,5]. Depending on the context and cell type, the tumor microenvironment may promote disease progression or, on the opposite, inhibit tumor growth. In this review, we review the different cell types in the tumor microenvironment and their interplay with neoplastic cells. A better understanding of the interactions between malignant cells and the tumor microenvironment could allow to design of new therapeutics targeting the microenvironment (Figure 2) and provide long-lasting disease control in mycosis fungoides and Sézary syndrome.

## 2. Overall T-helper Type 2 (Th2) Polarization of the Mycosis Fungoides/Sézary Syndrome Tumor Microenvironment

Early-stage mycosis fungoides is histologically difficult to distinguish from other inflammatory skin diseases, such as chronic eczema. The malignant cells of advanced-stage cutaneous T-cell lymphomas, albeit being highly heterogeneous [6,7], are generally Th-2 polarized [8]. In early lesions, an overall Th-1 polarization is observed in the skin lesions, with high expression of signal transducer and activator of transcription 4 (STAT4), a Th-1 marker [9], and a corresponding interferon-γ and tumor necrosis factor α signature [10,11,12]. Furthermore, patients with early-stage cutaneous T-cell lymphoma expressing Th2 markers (e.g., downregulation of STAT4, higher levels of IL-4) are more likely to progress and have reduced survival [13]. Advanced-stage cutaneous T-cell lymphoma displays a Th-2 polarization both in the skin tumor microenvironment [11] and blood [8]. The mechanisms triggering cutaneous T-cell lymphoma progression and this phenotypic switch are still unknown. Guenova et al. demonstrated that, in advanced cutaneous T-cell lymphomas, malignant lymphocytes are responsible for the major Th-2 polarization of the reactive infiltrate. In their absence, the reactive lymphocytes regain their unbiased status and produce interferon-γ instead of IL-4 [8].

Consequently, this Th-2 polarization was suggested as a possible therapeutic target in cutaneous T-cell lymphoma [10,14]. Dupilumab is a fully humanized monoclonal antibody against the alfa subunit IL-4 receptor, which blocks the signaling of key Th-2-cytokines IL-4 and IL-13, and is approved for the treatment of atopic dermatitis in adults. However, findings about the efficacy of dupilumab in cutaneous T-cell lymphomas are contradicting. While some reported beneficial effects [15] or complete remission at 2 months of treatment [16], others observed an exacerbation of the cutaneous T-cell lymphoma [17].

## 3. Targeting T-Cell Exhaustion via Immune Checkpoints

T cells can express different types of receptors that affect their behavior: costimulatory receptors that enable them to survive, proliferate, and resist programmed cell death, and co-inhibitory receptors that are linked to their functional exhaustion [18]. Increased expression of the immune checkpoint receptors Programmed cell death receptor 1 (PD-1), Cytotoxic T-Lymphocyte-Associated protein 4 (CTLA-4), Lymphocyte-activation gene 3 (LAG-3), and Inducible T-cell COStimulator (ICOS) on both CD4+ and CD8+ T cells have been observed in cutaneous T-cell lymphoma [19]. These findings support the use of immune checkpoint inhibitors to reverse T-cell exhaustion in the treatment of cutaneous T-cell lymphoma. 

### 3.1. The PD-1/PD-L1 and PD-L2 Axis

There is extensive research on the interactions between PD-1 and its ligands, PD-L1 and PD-L2. The PD-1 protein serves as a crucial regulator of self-tolerance by functioning as an immune checkpoint inhibitor. Its interaction with either of its ligands, programmed cell death ligand 1 (PD-L1) or PD-L2, leads to the suppression of T cell activation and function. Thus, the excessive expression of PD-L1 and PD-L2 by tumor cells can enable them to avoid attack by cytotoxic T cells [20]. In cancer, tumor-infiltrating T cells often express high levels of PD-1, which impairs their function and leads to T-cell exhaustion, while malignant cells may evade immune surveillance by expressing PD-L1 [21,22]. In cutaneous T-cell lymphomas, an additional layer of complexity is provided by the fact that malignant T cells themselves may express PD-1, and thus blockade of the PD-1/PD-L1 axis could potentially activate the malignant cells. Cutaneous T-cell lymphoma is characterized by the expression of PD-1 and its ligands on both tumor cells and cells of the microenvironment. Samini et al. used flow cytometry to analyze blood samples from cutaneous T-cell lymphoma patients and found that CD4+ T cells from patients with Sézary syndrome had significantly higher levels of PD-1 expression compared to those from patients with mycosis fungoides and healthy controls. They also observed that both CD26- and CD26+ populations of CD4+ T cells showed increased PD-1 expression when stimulated with anti-CD3/CD28 antibodies [23]. Similar findings were also observed by Cetinozman et al. in a study of patients with Sézary syndrome, where 89% (24 out of 27 cases) had more than 50% of their neoplastic T cells expressing PD-1 in their skin. In comparison, only 13% (8 out of 60) of patients with mycosis fungoides and 12% (1 out of 8) of patients with erythrodermic mycosis fungoides had more than 50% of their skin neoplastic T cells expressing PD-1 [24].

PD-1 expression has been implicated in both the development and progression of cutaneous T-cell lymphomas. In the development of cutaneous T-cell lymphomas, high levels of PD-1 expression have been observed on T cells within the skin, and this has been suggested to contribute to the immune escape and survival of the cancer cells [25]. Kantekure et al. found that PD-1 expression was commonly observed in the early stages of cutaneous T-cell lymphoma, specifically the patch and plaque stages, but was less frequent in advanced tumor stages. PD-L1 expression, on the other hand, seemed to increase as the disease progressed from early to advanced stages [26]. Regarding disease progression, Querfeld et al. conducted a case series study on 47 patients with cutaneous T-cell lymphoma, 57% of whom had stage IA-IIA disease and 43% had stage IIB-IVA2 disease, including seven of them with Sézary syndrome. All biopsies expressed PD-1, PD-L1, and ICOS, while PD-L1 was predominantly expressed on histiocytes/macrophages. There was a robust positive association between the expression of PD-L1 and both disease stage and large-cell transformation, which is consistent with the study by Kantekure et al. [26]. As previously reported [24], PD-1 was expressed in most cases of patients with Sézary syndrome [27]. By contrast with the results of Kantekure et al., there was no association between PD-1 expression and disease stage or the presence of large-cell transformation [27]. These discrepancies may be explained by the relatively low number of cases in these studies, directly linked to the rarity of the disease, by the potential heterogeneity of the cases, in particular the treatments received by the patients and staining protocols, and the inter-observer variability in the manual quantification of biomarkers, though the evaluation by two different pathologists may increase the reliability of the findings [27]. High expression of either ICOS, PD-1, or PD-L1 was observed in advanced-stage disease [27]. These findings suggest that PD-1, PD-L1, and ICOS may be involved in the development and progression of cutaneous T-cell lymphomas. However, the prognostic and predictive significance of PD-1 and PD-L1 expression in cutaneous T-cell lymphoma is not yet clear. According to a study by Park et al., where PD-1 was detected in 84% of mycosis fungoides cases and in 46% of other cutaneous T-cell lymphoma cases, there was no correlation between PD-1 expression and the course of mycosis fungoides [28].

Given its role in cutaneous T-cell lymphoma, the PD-1/PDL-1 axis has been studied as a target for cancer immunotherapy. This has led to clinical trials studying PD-1 (pembrolizumab/nivolumab) [29,30] and PD-L1 inhibitors (atezolizumab [31] and durvalumab [32]) in patients with cutaneous T-cell lymphoma. In a phase II clinical trial, pembrolizumab was administered to 24 patients with advanced and refractory cutaneous T-cell lymphoma. The treatment resulted in an overall response rate of 38%, including 2 complete responses and 7 partial responses. Of the 9 responding patients, 6 experienced a 90% or greater improvement in skin disease as measured by the modified Severity Weighted Assessment Tool (mSWAT). At the last follow-up, eight of the responding patients continued to show a response to treatment. The median duration of response could not be determined due to the median follow-up time of 58 weeks. In some cases, immune-related adverse events required the discontinuation of treatment. A temporary increase in erythroderma and itching was observed in 53% of patients with Sézary syndrome, but this did not lead to the cessation of treatment. This flare was found to be correlated with high PD-1 expression on Sézary cells but did not have any relation to the clinical response or lack thereof. There was no association between treatment outcomes and levels of PD-L1 expression, total mutation burden, or interferon-γ gene expression [29]. Additionally, nivolumab effectiveness has been shown to be lower in early studies and case reports, including a phase I trial with an overall response rate of 15% among 13 patients with mycosis fungoides [30].

Regarding PD-L1 inhibitors, a phase II trial evaluated atezolizumab in patients with stage IIb–IVB relapsed/refractory mycosis fungoides/Sézary syndrome [31]. The study found that 15.4% of patients with advanced stages of mycosis fungoides and Sézary syndrome, who were refractory or relapsed to prior systemic therapies, experienced a complete or partial response to treatment with atezolizumab, while 38.5% of patients had stable disease, 23.1% had progression, 11.5% experienced early death, and 11.5% were not evaluable. The median progression-free survival was 3 months, and the median time to the next systemic treatment was 5.9 months. The main reason for discontinuing treatment was disease progression. Additionally, a phase 1/2 clinical trial evaluated the anti -PDL1 durvalumab combined with the immunomodulator lenalidomide in patients with aggressive or refractory/advanced cutaneous T-cell lymphoma and determined the safety and efficacy of this regimen [32]. The study included 13 patients who were evaluated for toxicity and 12 patients who were evaluated for response. There were no serious adverse events or dose-limiting toxicities observed during the study. In terms of response, out of 12 patients evaluable for response, 9 achieved a partial response, 2 maintained stable disease, and 2 developed progressive disease. Of note, treatment responders had detectable levels of PD-L1 but low levels of ICOS versus high PD-L1 and ICOS levels in non-responders. 

Given the potential benefit of treating cutaneous T-cell lymphoma with PD-1 blockade but immune-related adverse events, it is important to identify predictors of response. In a study conducted by Philips et al., CO-Detection by indEXing (CODEX) and RNA sequencing were used to analyze tissue samples from 70 tumor regions in 14 patients with advanced cutaneous T-cell lymphoma who were participating in the above-mentioned pembrolizumab clinical trial. No differences were found in the frequencies of immune or tumor cells between patients who responded to treatment and those who did not. However, topographical variations were observed among effector PD-1+ CD4+ T cells, tumor cells, and immunosuppressive regulatory T cells (Tregs), resulting in the development of a spatial biomarker called the SpatialScore. This biomarker demonstrated a strong correlation with the response to pembrolizumab treatment in patients with cutaneous T-cell lymphoma. The SpatialScore was found to be correlated with variations in the functional immune state of the tumor microenvironment, T cell function, and the recruitment of chemokines specific to tumor cells. This was confirmed using a simplified tissue imaging platform that is easily accessible in the clinical setting [33]. Finally, cases of disease hyperprogression after PD-1/PD-L1 blockade have been reported in various types of tumors and have been associated with poor outcomes [34]. Recently, Gao et al. reported the development of disease hyperprogression in a patient with cutaneous T-cell lymphoma after PD-1 blockade, presumably due to the release of oncogenic T-cell receptor signaling with the removal of PD-1 suppression. Functional PD-1 was expressed at the surface of malignant T cells. Somatic amplification of PRKCQ (encoding PKCθ, a key player in the T cell activation/NF-kB pathway) was found in the malignant T cells by single-cell RNA sequencing [35]. Such findings highlight the potential need for tumor molecular profiling that could identify patients at risk of hyperprogression.

### 3.2. ICOS/ICOS-L

ICOS is a key costimulatory receptor, a member of the CD28/B7 superfamily, that plays a crucial role in enhancing T-cell activity. It is expressed at low levels on naive T cells, but its expression is quickly induced upon T-cell receptor engagement. ICOS has a unique ligand, ICOSL, which is expressed by a variety of cells, including antigen-presenting cells, and their engagement leads to a positive co-stimulatory signal [36]. 

The engagement of the ICOS receptor can have different results based on the involved T-cell subset. For example, ICOS/ICOS-L binding results in an antitumor response by CD8+ cytotoxic T lymphocytes (CTL), CD4+ Type 1 helper T cells (Th1), and follicular helper T cells (Tfh). However, activation of ICOS/ICOS-L may also result in a pro-tumor response, mainly mediated by Treg cells. Thus, both anti-ICOS agonists and antagonists are investigated in different cancers [37].

Specifically for cutaneous T-cell lymphoma, when Phillips et al. developed and applied a 56-marker CODEX antibody panel to eight cutaneous T-cell lymphoma samples, they found that ICOS was strongly expressed on CD4+ T cells and Tregs [38], while Geskin et al. observed that Tregs from patients with SS markedly suppressed proliferation of autologous CD4+CD25- responder T cells [39]. Additionally, Amatore et al. provided valuable insight into the role of ICOS in cutaneous T-cell lymphoma [40]. Immunohistochemistry analysis revealed that ICOS protein was present in the majority of 52 skin biopsy samples from individuals with mycosis fungoides and Sézary syndrome. The protein was also detected in biopsy samples from lymph nodes of individuals with Sézary syndrome. Flow cytometry analysis showed that circulating tumor cells in all patients with Sézary syndrome strongly expressed ICOS. The percentage of ICOS-positive Treg cells was significantly higher in these patients compared to healthy donors. The above data suggest that the upregulation of costimulatory receptors such as ICOS on the surface of malignant T cells may be involved in the development of SS, and the ICOS-ICOS ligand costimulatory pathway may be a promising target in cutaneous T-cell lymphoma immunotherapy [37]. 

To summarize, in cutaneous T-cell lymphomas, the overexpression of ICOS by malignant T cells and Treg cells suggests that depletion of ICOS-expressing cells could both directly reduce the tumor cell burden and activate the antitumor immune responses by killing Tregs. 

Amatore et al. also investigated the potential of anti-ICOS antibody-drug conjugates (ADCs) as a treatment for cutaneous T-cell lymphoma. These drugs were created by combining murine anti-ICOS antibodies with monomethyl-auristatin E (MMAE) and pyrrolobenzodiazepine. A decrease in the viability of three cutaneous T-cell lymphoma cell lines—Myla, MJ, and HUT78—was observed in response to treatment with anti-ICOS ADCs. This decrease was found to be dependent on the dosage of the drug. It is worth mentioning that anti-ICOS-MMAE ADCs were also more effective than brentuximab vedotin, an anti-CD30-targeting ADC, in an in vivo xenograft mouse model. The evaluation of the effectiveness of anti-ICOS ADCs in patient-derived xenografts from individuals with SS and angioimmunoblastic T-cell lymphoma yielded promising results [40].

Although promising, in vivo human data on therapies targeting the ICOS/ICOS-L pathway cutaneous T-cell lymphoma are still limited. In a phase I trial of anti-ICOS antibody for relapsed/refractory cutaneous T-cell lymphoma, peripheral T-Cell lymphoma, and angioimmunoblastic T-Cell lymphoma, anti-ICOS antibody MEDI-570 was safe, well tolerated, and showed promising clinical activity in poor-risk refractory angioimmunoblastic T-Cell lymphoma. In that phase 1 trial, there were only 2 patients with cutaneous T-cell lymphoma [41].

### 3.3. TIGIT

The TIGIT receptor is a protein that is expressed on immune cells such as CD4+ and CD8+ T cells, Tregs, and natural killer cells. TIGIT, along with CD96, are co-inhibitory receptors that work along with CD226 to create a pathway similar to the CD28/CTLA-4 [42]. 

The deficiency of TIGIT in mice leads to uncontrollable T-cell responses and suppressed tumor growth. On the other hand, overexpression of TIGIT on tumor-infiltrating lymphocytes is associated with advanced stages of cancer. This may be attributed to the downregulation of Th1 and Th2 cells and the limitation of CD8 T cell responses [43]. In mice with a heavy tumor burden, blockade of the TIGIT pathway prevented NK cell exhaustion and promoted NK-cell-dependent tumor immunity [44]. These findings suggest a critical role for TIGIT in modulating immune responses and highlight its potential as a therapeutic target in the autoimmune and cancer contexts. 

Anzengruber et al. found that compared to healthy controls, tumor cells from Sézary syndrome patients had significantly higher expression of BTLA, FCRL3, and TIGIT, while LAG-3 expression was reduced. Subtle differences were observed in CTLA-4 expression [45]. Preillon et al. conducted a study examining TIGIT expression in healthy donors and cancer patients. They discovered that TIGIT was present on tumor cells in hematologic cancers such as cutaneous T-cell lymphoma [46]. Tregs expressed the highest density of the TIGIT receptor. In cancer patients and mice, high TIGIT expression in Tregs was correlated with effective antibody-dependent killing and preferential depletion of the immunosuppressive Treg population.

In the CITYSCAPE phase II clinical trial (NCT03563716), the combination of tiragolumab, a novel anti-TIGIT inhibitory immune checkpoint agent, and atezolizumab (an anti-PD-L1 antibody) showed a clinically significant improvement in progression-free survival (5.4 months compared to 3.6 months) and a higher overall response rate (31.3% compared to 16.2%) compared to atezolizumab and placebo in chemotherapy-naive, PD-L1-positive recurrent or metastatic non-small cell lung cancer patients [47]. Anti-TIGIT antibody efficacy is yet to be evaluated in patients with cutaneous T-cell lymphoma. 

## 4. Chemokine Receptors

### 4.1. CCR4

CCR4 is a chemokine that is overexpressed by type 2 helper T cells in Tregs and certain tumor lymphocytes, such as cutaneous T-cell lymphoma tumor cells. CCR4 is involved in T-cell skin tropism [48].

CCR4 is consistently overexpressed in mycosis fungoides and Sézary syndrome, and a high number of CCR4-positive T cells has been associated with poor prognosis [49]. CCL22 and CCL17 are ligands for the CCR4 protein. This interaction is essential in cutaneous T-cell lymphoma pathogenesis, as it allows CCR4-positive tumor T cells homing to the skin and attracts CCR4-positive Tregs to the tumor microenvironment.

Mogamulizumab (KW0761), a humanized monoclonal antibody, was designed to specifically inhibit the activity of the CCR4 protein and deplete CCR4-expressing cells. In the MAVORIC study, mogamulizumab was associated with longer progression-free survival compared to vorinostat in previously treated cutaneous T-cell lymphoma [50]. De Masson et al. reported a study of 44 patients with cutaneous T-cell lymphoma who were treated with mogamulizumab and in which 32% of patients developed skin rashes. Mogamulizumab-associated rashes were associated with significantly higher overall survival rates (hazard ratio, 0.16; 0.04–0.73; *p* = 0.01) and longer time to disease progression in patients with Sézary syndrome. The rashes were histologically characterized by a granulomatous and/or lichenoid skin infiltrate that expressed the CD163+ and CD8+ markers, respectively. Analysis of T-cell receptor β genes in both skin and blood samples using high-throughput sequencing revealed the depletion of cutaneous T-cell lymphoma cells and the recruitment of new, reactive T-cell clones in the skin at the time of the rash. Additionally, the skin rashes exhibited a Th-1 polarisation, an overexpression of CXCL9 and CXCL11, which are macrophage-derived chemokines that attract CXCR3+ T cells to the skin. At baseline, patients with rashes had a higher frequency of TIGIT+ and PD-1+ exhausted reactive blood T cells, which decreased after treatment with mogamulizumab [51].

This suggested that mogamulizumab may be effective in achieving long-term immune control of cutaneous T-cell lymphoma in patients with rash by activating macrophages and T-cell responses. The data also highlighted the important role of macrophages and CD8 T cells in the skin response to therapeutic monoclonal antibodies in cutaneous T-cell lymphoma.

### 4.2. CCR8

Loss of CCR4 expression by tumor T cells can appear after mogamulizumab therapy [52,53]. However, there are other chemokine receptors, such as CCR8 (CD198), that also facilitate T-cell homing to the skin. CCR8 is expressed by skin resident memory T cells, which are thought to be the tumor cell of origin in mycosis fungoides [54]. Additionally, CCR8 is highly expressed by tumor-infiltrating Tregs that contribute to immune evasion, while the expression on peripheral blood Treg is lower [55].

In mouse models of colon, melanoma, breast, and urothelial cancer, research has shown that tumor-associated Tregs expressed high levels of CCR8. When targeting CCR8 with specific antibodies in monotherapy, there was a significant reduction in tumor growth, similar to what has been observed with the use of anti-PD-1 blocking antibodies. These preclinical findings suggest that targeting CCR8 may be a promising approach for the treatment of cancer. Depleting CCR8+ Tregs showed a strong antitumor effect on its own or in combination with a PD-1 inhibitor in mouse models of cancer [56]. Unlike CCR4, the target of mogamulizumab, CCR8 was selectively expressed on tumor Tregs and minimally expressed on effector T cells [55].

Giustiniani et al. were the first to explore the potential of targeting CCR8 for the treatment of cutaneous T-cell lymphoma. In this study, they analyzed the peripheral blood of 13 patients with SS and persistent blood involvement and found that Sézary cells in the blood of cutaneous T-cell lymphoma patients express higher levels of the homing marker CCR8 compared to healthy controls’ T cells. The chemokines CCL8 and CCL18, which are known to bind CCR8, were found to be highly expressed in the skin of cutaneous T-cell lymphoma patients and may play a role in the homing of CCR8-expressing lymphocytes to the skin in these patients [57].

Since CCR8 was shown to be expressed by tumor cells in cutaneous T-cell lymphoma and by tumor-infiltrating Tregs in tumors, targeting CCR8-expressing cells may be a useful strategy to treat cutaneous T-cell lymphoma. It may help to eradicate tumor cells and stimulate an immune response against T-cell lymphoma cells. 

## 5. Role of the Connective Tissue Cells and Other Cells of the Tumor Microenvironment in Mycosis Fungoides and Sézary Syndrome

### 5.1. Keratinocytes, Corneocytes, and the Microbiome

The role of the microbiome in mycosis fungoides and Sézary syndrome was thoroughly reviewed by Joost and Wehkamp in a previous issue [58]. The epidermotropism of the lymphocytes suggests the role of keratinocytes in the tumor microenvironment (Figure 1). In early-stage mycosis fungoides, keratinocytes express high levels of CXCL9 and CXCL10 to help CD8 lymphocyte recruitment [12]. As feedback, benign T cells in cutaneous T-cell lymphoma express interleukin 22 (Il-22), which may foster the expression of chemokine ligand 20 (CCL20) expression via STAT3 activation [59]. This leads to epidermal hyperplasia, a feature that is frequently observed in mycosis fungoides. The Th-2 mediators periostin (POSTN), IL-4, and IL-13 were shown to stimulate keratinocytes to express increased levels of IL-25, which promotes Th2 polarization [60,61]. The higher Il-25 expression in keratinocytes from cutaneous T-cell lymphoma skin enhances the expression of IL-13 in IL-25 receptor-positive malignant T cells through the activation of STAT6 [61]. On the other hand, by producing antimicrobial peptides, the keratinocytes may alter the microbiome, which may stimulate the malignant cells [58].

### 5.2. Fibroblasts

In addition, members of the non-lymphocytic tumor microenvironment were shown to play a major role in cutaneous T-cell lymphoma pathogenesis. Takashi et al. [62] showed the involvement of fibroblasts in creating a Th-2 polarized microenvironment by producing periostin when stimulated by IL-4/IL-13. Periostin leads to TSLP production by keratinocytes, while IL-4 induces IL-25 production by keratinocytes. The presence of TSLP and IL-25, on the other hand, boosts Th-2 cytokines and Th-2 proliferation, creating an amplification loop [62]. Hodak et al. [63] highlighted the role of CXCL12, produced by the cutaneous T-cell-lymphoma-associated fibroblasts, in malignant lymphocyte attraction and chemoresistance of malignant lymphocytes. He et al. [64] reported the key role of fibroblast subpopulations in maintaining the Th-2 microenvironment in atopic dermatitis by using a recent technique. Additionally, the presence of macrophages with an M2 phenotype was described as cutaneous T-cell lymphoma, which produces the chemokines CCL18 and CXCL10 [10,11,65]. Our previous findings indicate that other members of the non-lymphocytic tumor microenvironment may orchestrate the inflammatory response [66] and drive the inflammation. We observed, for instance, the CXCL9-CXCL10 production by keratinocytes and macrophages in the cutaneous T-cell lymphoma microenvironment [66]. However, the precise cell–cell interactions are not yet fully understood. It is unclear what causes the phenotypic switch from a Th-1 to a Th-2 polarization in cutaneous T-cell lymphoma.

### 5.3. Endothelial Cells

Endothelial cells are present in the tumor microenvironment of mycosis fungoides and Sézary syndrome as lymphangial cells, arterial and venular cells. High expression of endothelial and lymphatic markers such as podoplanin may contribute to malignant cell proliferation and expansion, as reflected by the reduced overall survival of patients with higher expression of these markers [67,68]. Additionally, the expression of vascular endothelial growth factors A and C (VEGF-A, VEGF-C) was demonstrated in the skin by malignant T cells [68,69,70]. The expression of matrix metalloproteases (MMPs) by the malignant cells and fibroblasts may further contribute to angiogenesis [71].

### 5.4. Macrophages

Macrophages are one of the most abundant cell types in the microenvironment of mycosis fungoides and Sézary syndrome [66]. While antitumorigenic M1 macrophages produce Th-1-associated cytokines, the protumorigenic M2 macrophages are better characterized in cutaneous T-cell lymphomas. CD163, a marker of M2 macrophages, is associated with a worse prognosis [72,73]. Stimulation of the macrophages by periostin (that is produced mainly by fibroblasts) strengthens M2 differentiation [73,74]. Consequently, macrophages are being targeted by novel immunotherapies. One promising strategy is the inhibition of the CD47-SIRPα interaction via anti-CD47 antibodies [75]. This increases antitumor immune responses by macrophages in the tumor microenvironment by suppressing the “don’t eat me” signal given by tumor cells to macrophages [76]. Found on both dendritic cells and macrophages, CD40 was also recently proposed as a treatment target [65]. 

### 5.5. Dendritic Cells

Closely related to macrophages, dendritic cells (DCs) originate from circulating monocytes. Three subtypes may be present in the skin, Langerhans cells, dermal DCs, and plasmacytoid DCs. Langerhans cells were shown to organize the Pautrier’s microabscesses [77]. DCs, as a group of antigen-presenting cells, may drive skin inflammation in cutaneous T-cell lymphomas and could be the target of future therapies. Clark et al. suggested CD40, OX40, and c-KIT as promising therapeutic avenues in mycosis fungoides [65]. 

### 5.6. Mast Cells

Mast cells are the main promoters of itching in the skin. Through clinical observations and experimental data, Rabenhorst et al. suggested the potential role of mast cells in tumorigenesis [78]. Later research pointed to their key role in especially early stages of mycosis fungoides [79]. However, using single-cell sequencing, the group of Geskin et al. demonstrated the presence of mast cells also in advanced-stage cutaneous T-cell lymphoma [7].

## 6. Conclusions

Cutaneous T-cell lymphomas are rare non-Hodgkin lymphomas with a median overall survival lower than 5 years in the advanced stage and usually short-lasting responses to therapy. New therapeutic approaches are a highly unmet clinical need, and a better understanding of the role of reactive immune cells in the tumor microenvironment may enable to enhance of antitumor immune responses. Patients with cutaneous T-cell lymphoma treated with mogamulizumab, an anti-CCR4 depleting antibody, and immune-mediated skin rashes had a completely modified immune microenvironment at the time of rash, better treatment responses, and longer overall survival, suggesting that modulation of the immune microenvironment is key in achieving long-term disease control. Immune checkpoint inhibition to activate exhausted nonmalignant T cells (PD-1/PD-L1 axis) or modulation of the CD47-SIRPalpha axis to promote macrophage antitumor immune responses are other promising novel therapies of cutaneous T-cell lymphomas. New techniques such as single-cell RNA sequencing or multiplex immunofluorescence and imaging mass cytometry, the two latter allowing the precise visualization of the spatial interactions between tumor cells and cells of the microenvironment, will lead to new steps in the understanding of the complex relationship between malignant and nonmalignant cells in cutaneous T-cell lymphomas and provide new therapeutic approaches.
